# Synthesis, characterization and crystal structures of platinum(II) saccharinate complexes with 1,5-cyclooctadiene

**DOI:** 10.3906/kim-2002-34

**Published:** 2020-06-01

**Authors:** Ceyda İÇSEL

**Affiliations:** 1 Department of Chemistry, Faculty of Arts and Sciences, Bursa Uludağ University, Bursa Turkey

**Keywords:** Pt(II) complex, saccharinate, 1,5-cyclooctadiene, crystal structure

## Abstract

Two new platinum(II) complexes, namely [PtCl(sac)(COD)] (1) and [Pt(sac)_2_(COD)] (2) (sac = saccharinate and COD = 1,5-cyclooctadiene), were synthesized and characterized by elemental analysis, IR, NMR, ESI-MS spectroscopic and thermal analysis (TG/DTA) methods. The platinum(II) complexes were prepared from the reaction of [PtCl_2_(COD)] with Na(sac)•2H_2_O. The addition of the sac ligand resulted in the replacement of 1 and 2 chlorido ligands in [PtCl_2_(COD)] to yield 1 and 2, respectively. The structures of the complexes were determined by single crystal X-ray diffraction and showed a distorted square planar coordination geometry around platinum(II). COD acted as a π-donor ligand, while sac was N -coordinated in both complexes. The TG/DTA data indicated that both complexes were thermally stable up to 220 °C in air and their thermal decompositions yielded Pt as a final product. Complexes 1 and 2 were also designed as possible precursors to synthesize new mixed-ligand platinum(II) sac complexes in a one-pot reaction.

## 1. Introduction

After the discovery of the cell growth inhibition effect of cisplatin by Rosenberg [1–3], the design and synthesis of new platinum(II) complexes received increasing attention. For this purpose, several synthetic routes were used. One way is to synthesize precursors to prepare new platinum(II) complexes. The reaction of a simple platinum(II) complex (K_2_[PtCl_4_]) with the mono-, di- or tridentate ligands usually leads to the formation of chloridoplatinum(II) complexes bearing these ligands. In a subsequent reaction, the chlorido ligands in the precursor can be exchanged by another ligand to yield the desired platinum(II) complex. Another alternative way is to use [PtCl_2_(COD)] (COD =1,5-cyclooctadiene), which serves as an excellent starting complex for a wide range of organoplatinum(II) compounds [4]. The chlorido ligands in [PtCl_2_(COD)] can be replaced by other higher halides (Br^-^ or I^-^) or anionic organic ligands due to the high *π trans* effect of COD [5]. Organoplatinum(II) complexes [Pt(R)(L)(COD)] (R = organic groups such as alkyl, aryl or alkynyl; L = other ligands) received great interest and were widely used in catalysis [6–10], material science [11,12] and medicinal research as anticancer agents [13–19].

The artificial sweetener saccharin (sacH, 1,1-dioxo-1,2-benzothiazol-3-one) loses its imine hydrogen in solution and the corresponding saccharinate (sac) anion acts as a multifunctional ligand towards various metal ions [20]. It forms coordination compounds from mononuclear to polynuclear. Among the metal complexes of sac, the platinum(II) complexes of sac with different ligands particularly seem to be promising anticancer agents [21–29]. In the synthesis of these complexes, a variety of synthetic strategies was applied. One efficient route was the progressive replacement of the chlorido ligands in [PtCl_2_(COD)] by the sac ligand to obtain the first examples of organometallic platinum(II) sac complexes, [PtCl(sac)(COD)] (1), and [Pt(sac)_2_(COD)] (2). In this study, the synthesis, characterization and crystal structures of 1 and 2 were described. Both complexes can also be used as precursors for the preparation of new platinum(II) sac complexes. The COD ligand in 1 and 2 can further be exchanged by σ-donor neutral ligands to give novel mixed-ligand chlorido platinum(II) sac or bis(sac)platinum(II) complexes. The synthetic method seems to be an attractive alternative way to synthesize new platinum(II) sac complexes, because it may be regarded as a one-pot reaction with high yields. However, the other procedures include multistep synthesis and usually result in low yields.

## 2. Results and discussion

### 2.1. Synthesis and characterization

[PtCl(sac)(COD)] (1) and [Pt(sac)_2_(COD)] (2) were designed as starting materials for the synthesis of new mixed-ligand platinum(II) complexes of sac. 1 and 2 were synthesized by the reaction of [PtCl_2_(COD)] with Na(sac)•2H_2_O in a molar ratio of 1:2 and 1:5, respectively. Both complexes were obtained in high yields over 90%. They are stable in air at room temperature (rt) and soluble in MeCN, MeOH, EtOH, DMSO, and DMF as well as in the aqueous solutions of these solvents. The conductivity measurements at rt indicated that both complexes are nonelectrolyte in MeOH.

The structures of 1 and 2 were confirmed by a number of spectroscopic methods. The IR spectra of the complexes present the characteristic absorption bands of both COD and sac ligands. The weak bands centred at 3008 and 3077 cm^-1^ are assigned to the C=CH vibrations of COD and sac, respectively, while the aliphatic CH bands of COD occur between 2919 and 2962 cm^-1^. The strong bands at 1673 and 1691 cm^-1^ are attributed to the carbonyl group of sac in 1 and 2, respectively. The asymmetric and symmetric stretching of the sulfonyl group of sac appear as very sharp bands at ca. 1302/1285 and 1172/1155 cm^-1^, respectively. Moreover, the weak band centred at 1335 cm^-1^ is due to the symmetric vibration the CNS moiety of sac and the asymmetric mode of this group is observed at ca. 970 cm^-1^ as a strong band.

**Figure 1:**
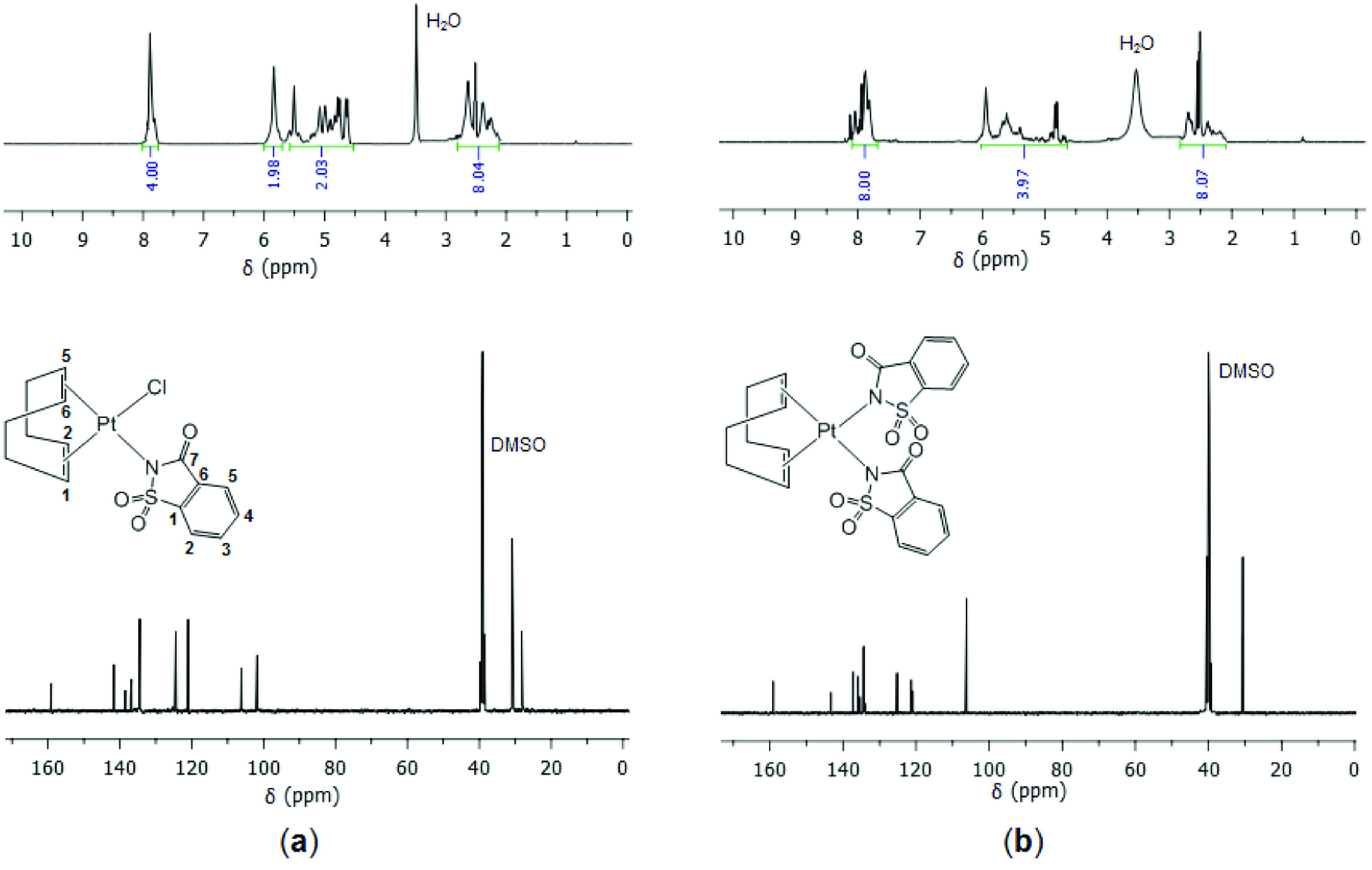
^1^H and ^13^C{^1^H}NMR spectra of 1 (a) and 2 (b).

The 1 H NMR spectra in Figure 1 display the phenyl protons of sac in the range of 7.94–7.73 ppm. The 2 =CH protons of COD in 1 appear as 2 signals at 5.84 and 5.08 ppm, while those in 2 occur at 5.52 ppm. The notable difference between the 2 olefinic chemical shifts in 1 is due to the trans influence of the chlorido and sac ligands. On the other hand, a COD ligand together with 2 sac ligands in 2 results in only 1 signal for the 2 equivalent =CH protons. The splitting of these signals is evident because of coupling with the Pt nucleus. The ^2^J_Pt,H_(=CH, COD) coupling constants are found as 36 and 64 Hz for 1 and consistent with the somewhat higher trans influence of sac [15,29]. The corresponding coupling constant is approximately 40 Hz for 2. In addition, the signals between 2.78 and 2.08 ppm are assigned to the methylene protons of COD. The ^13^C{^1^H}NMR spectra of both complexes exhibit the expected number of signals. The carbonyl signal of sac appears at 159.1 and 158.9 ppm for 1 and 2, respectively. The phenyl C atoms are observed in the range of 141.7–121.4 ppm for 1, and 141.7–121.0 ppm for 2. Complex 1 gives rise to 2 olefinic C signals at 106.2 and 101.8 ppm and 2 methylene C signals at 30.7 and 27.8 ppm, while the corresponding C atoms in 2 resonance at 106.1 ppm and 30.4 ppm.

The ionization of the complexes in MeOH was investigated by electrospray mass spectroscopy. The positive ion electrospray ionization (ESI^+^) mass spectra of 1 present the following ions [M + 1]^+^ (22%; m/z = 522.2), [Pt(sac)(MeOH)]^+^ (100%; m/z = 408.4) and [Pt(sac)]^+^ (32%; m/z = 375.5), while 2 forms [M + Na]^+^ (39%; m/z = 691.4), [Pt(sac)(MeOH)]^+^ (63%; m/z = 408.4) and [Pt(sac)]^+^ (100%; m/z = 375.5) ions in solution under the experimental conditions. The molecular ion peaks of 1 and 2 are apparent in the spectra as the [M + 1]^+^ ion (m/z 522.2) and the sodium adduct, [M + Na]^+^ (m/z 691.4), respectively, while the base peaks (100%) correspond to the [Pt(sac)(MeOH)]^+^ ion at m/z 408.4, and the [Pt(sac)]^+^ ion at m/z 375.5 in the spectra of 1 and 2, respectively.

## 3. Structures of the platinum(II) complexes

**Figure 2:**
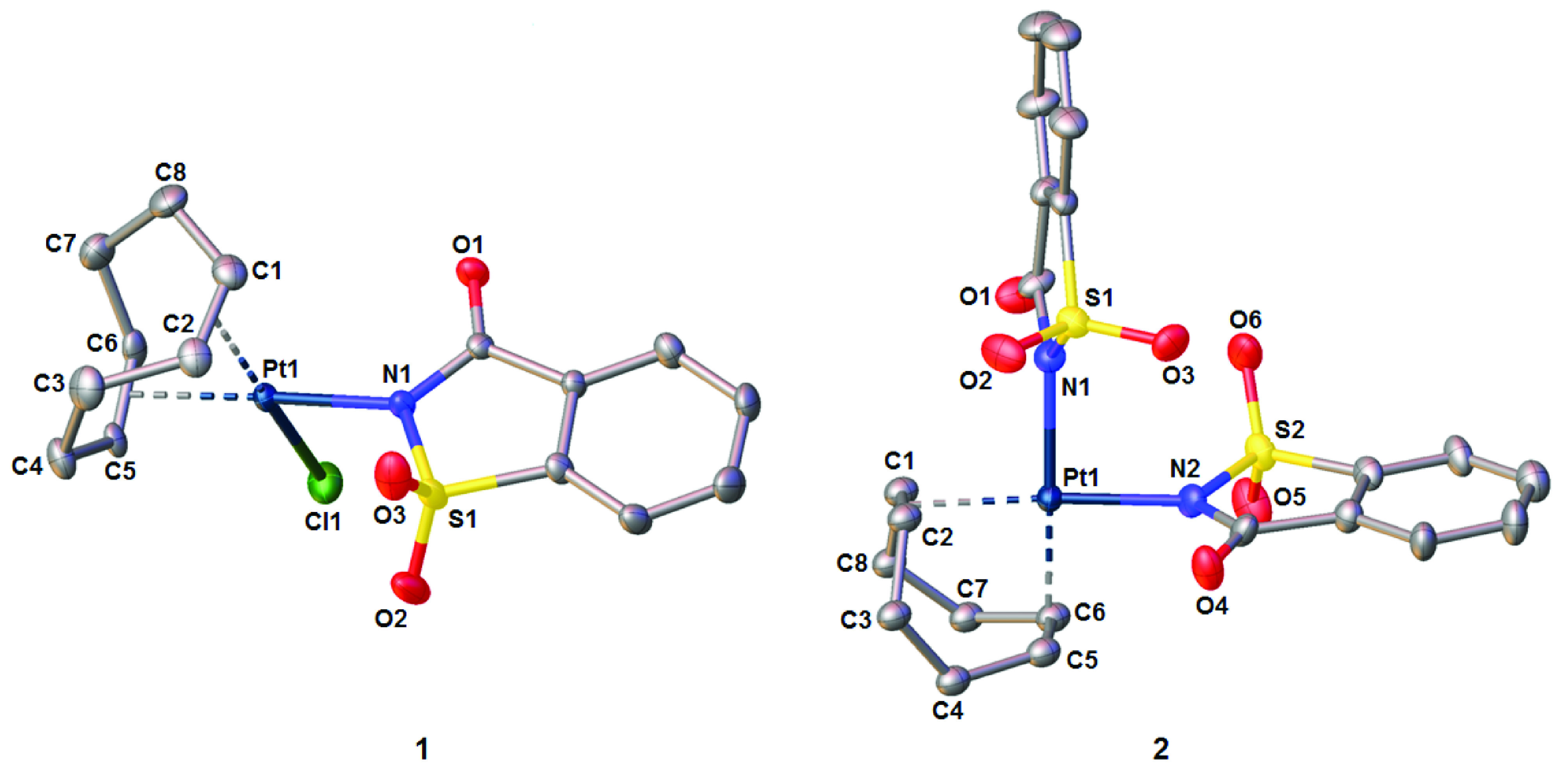
Molecular structures of 1 and 2. Hydrogen atoms were omitted for clarity.

Slow evaporation of the saturated solutions of 1 and 2 at rt afforded white prisms. Complexes 1 and 2 crystallize in the monoclinic space group P2_1_/c and orthorhombic space group *Pna* 2_1_, respectively. The molecular structures of both complexes were given in Figure 2, while selected bond lengths and angles are given in Table 1. In both complexes, the platinum(II) ion shows a distorted square planar coordination geometry defined by the mid-points of the 2 π-coordinated double bonds of COD, 1 chloride and 1 N -bonded sac anion in 1, and 2 N -coordinated sac ligands in 2 (Figure 2). In 1 and 2, the COD ligand coordinates to the platinum(II) ion in η^4^-type coordination and in the boat conformation with the double-bond lengths of ca. 1.38 Å. The Pt–C bond distances lie within the typical range for the platinum(II) complexes of COD [17,30,31]. However, 2 sets of the Pt–C bonds were observed in 1, most likely due to the different trans influences of the chlorido and sac ligands. In addition, 1 of the 4 Pt–C bonds in 2 is notably shorter than the rest and consequently, the molecule retains a pseudo C2 symmetry in the solid state. This is consistent with the similar observations reported for [Pt(COD)Me_2_] [32]. The sac ligand is coordinated to platinum(II) through the negatively charged N atom. The 2 sac ligands in 2 are oriented almost perpendicularly with a dihedral angle of 86.15(6)° to reduce the steric hindrance. The Pt–N bond distances in both complexes are similar and compare well with the corresponding bonds in the platinum(II) complexes with sac [22–29,33]. On the other hand, the Pt–Cl bond distance of 2.301(2) Å in 1 is somewhat shorter than those in cis-[PtCl(sac)(L)_2_] [26,29, 33] and [PtCl(COD)Me] [32].

**Table 1:** Selected bond lengths (Å) and bond angles (°) of 1 and 2.

	**1**	**2**
Pt-Cl1	2.301(2)	-
Pt-N1	2.041(4)	2.054(14)
Pt-N2	-	2.006(14)
Pt-C1	2.142(6)	2.177(18)
Pt-C2	2.158(6)	2.180(20)
Pt-C5	2.172(4)	2.173(17)
Pt-C6	2.182(4)	2.134(19)
Pt-X1^a^	2.033	2.071
Pt-X2^a^	2.062	2.039
N1-Pt-C11	89.89(13)	-
N1-Pt-N2	-	90.4(6)
N1-Pt-C1	90.00(19)	91.8(6)
N1-Pt-C2	92.76(18)	92.6(7)
N1-Pt-C5	158.73(15)	165.7(9)
N1-Pt-C6	163.82(16)	156.9(8)
N2-Pt-C1	-	166.5(8)
N2-Pt-C2	-	156.8(7)
N2-Pt-C5	-	91.2(7)
N2-Pt-C6	-	89.4(7)
Cl1-Pt-C1	158.06(14)	-
Cl1-Pt-C2	163.93(14)	-
Cl1-Pt-C5	90.51(16)	-
Cl1-Pt-C6	92.54(17)	-
C1-Pt-C5	97.4(2)	89.9(7)
C1-Pt-C6	81.8(2)	83.3(7)
C2-Pt-C5	81.3(2)	80.4(7)
C2-Pt-C6	89.3(2)	96.6(7)

^a^ X1 and X2 refer to the centroids in the middle of olefinic bonds C1–C2, and C5–C6, respectively.

The discrete molecules of 1 and 2 are held together in the solid state by a number of weak C–H•••O hydrogen bonds involving the carbonyl or sulfonyl oxygen atoms of sac. Interestingly, no Cl•••H interactions are observed in 1.

## 4. Thermal analysis

**Figure 3:**
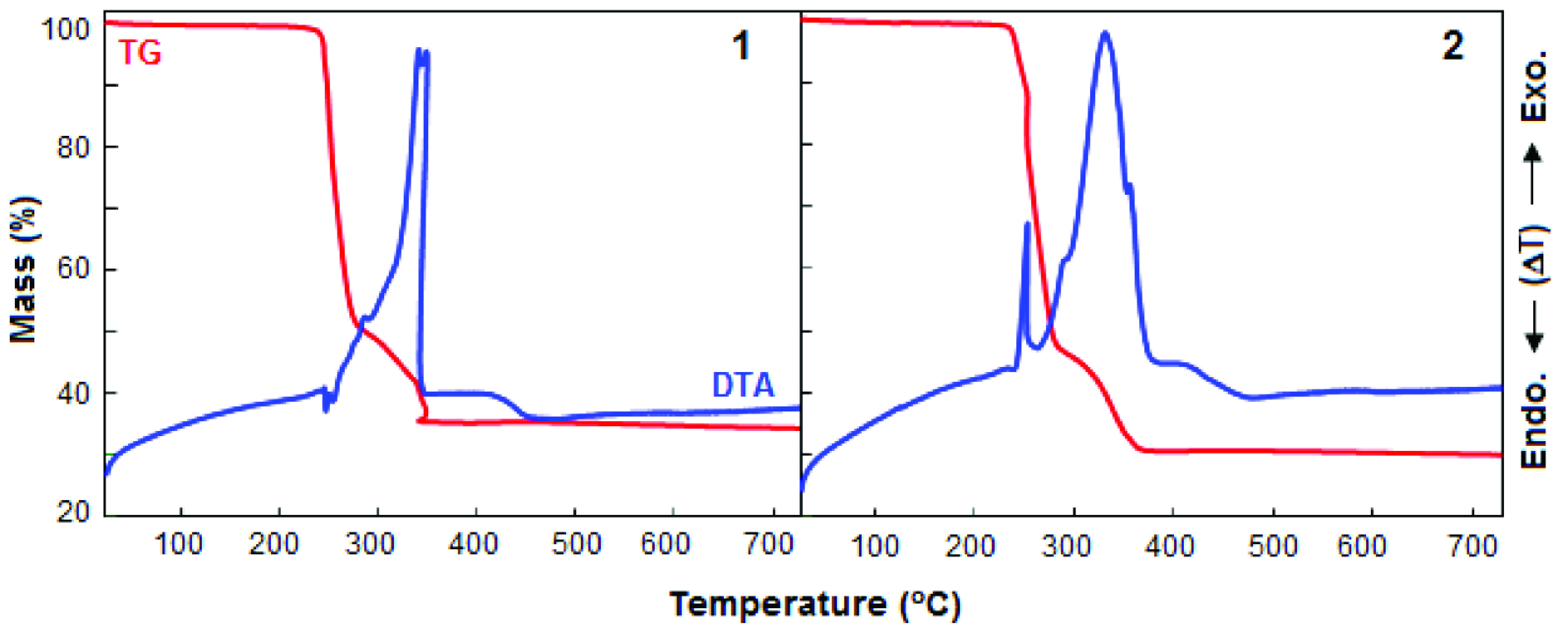
Thermal analysis curves of 1 and 2.

The thermal stability and decomposition behaviour of the complexes were studied by TG and DTA in air. As shown in Figure 3, complexes 1 and 2 are thermally stable up to ca. 220 °C. 1 decomposes in 2 steps, which do not correspond to the removal of any species. Its DTA curve presents a small endothermic and a highly exothermic effects centred at 250 and 340 °C, respectively. The latter is likely to due to combustion of the sac moiety. The decomposition of this complex ends at 360 °C to give metallic Pt as a residue (found: 36.2% and calcd: 37.5%). On the other hand, complex 2 also exhibits a 2-stage exothermic decomposition centred at 253 and 331 °C in the DTA curve. The decomposition of 2 also yields metallic Pt as an end product (found: 30.6% and calcd: 29.2%) at 390 °C.

## 5. Conclusions

Two new platinum(II) complexes containing COD and sac ligands were successfully synthesized via the progressive replacement of the chloride ligands in [PtCl_2_(COD)] by the sac ligand. Both complexes were characterized by spectroscopic (IR, NMR and ESI-MS) and thermal methods (TG/DTA). Their crystal and molecular structures were determined using X-ray crystallography. The platinum(II) ion in both complexes has a distorted square-planar coordination geometry mainly due to coordination of the chelating COD ligand. [Pt(sac)_2_(COD)] (2) has approximate C2 symmetry, which results in a signal for the olefinic proton and carbon atoms in ^1^H and ^13^C spectra, whereas [PtCl(sac)(COD)] (1) gives 2 signals in the NMR spectra for the corresponding groups due to the difference in the trans effects of the chlorido and sac ligands. The complexes exhibit high thermal stability in air. Finally, both complexes can be considered as precursors for the synthesis of new mixed-ligand platinum(II) sac in a one-pot reaction, in which COD in the complexes is readily replaced by the other ligands.

## 6. Experimental

### 6.1. Materials and measurements

[PtCl_2_(COD)] was prepared according to the reported methods [4]. Elemental analyses for C, H, and N were performed using a Costech elemental analyser. FTIR spectra were recorded on a Perkin Elmer Spectrum Two FT-IR spectrophotometer in the frequency range 4000-400 cm^-1^. NMR spectra were obtained using a Bruker Avance III (400.13 MHz ^1^H and 100.62 MHz ^13^C) NMR spectrometer in DMSO-d_6_. ESI-MS spectra were recorded using a Bruker Daltonics Microtof II-ESI-TOF mass spectrometer. Thermal analysis curves (TG and DTA) were obtained from a Seiko Exstar TG/DTA 6200 thermal analyser in a flowing atmosphere of air with a heating rate of 10 K min^-1^ using a sample size of ca. 10 mg and platinum crucibles. The electrical conductivity measurements of the complexes in MeOH were carried out with HANNA HI 5521 at rt. Melting points are measured using a BUCHI 560 instrument.

### 6.2. Synthesis of the platinum(II) complexes

#### 6.2.1. Synthesis of [PtCl(sac)(COD)] (1)

A solution of [PtCl_2_(COD)] (0.25 mmol, 94 mg) in CH_2_Cl_2_ (10 mL) was added to a solution of Na(sac)•2H_2_O (0.50 mmol, 121 mg) in MeOH (20 mL). The resulting solution was refluxed at 70 °C for 24 h and then the solvent was removed using a rotary evaporator to yield a white powder. The powder was washed with water twice and dried in air. Single crystals of the complex were obtained by slow crystallization from a mixture of EtOH/H_2_O/DMF within 4 weeks at rt.

[PtCl(sac)(COD)] (1). White powder. Yield: 117 mg (90%). Mp: 246–251 °C (decomp.). Anal. calcd. for C_15_H_16_ClNO_3_ PtS: C, 34.59; H, 3.10; N, 2.69%. Found C, 34.92; H, 3.27; N, 2.48%. IR (ν /cm^-1^): 3076w, 3025w (C=CH), 2962w, 2927w (CH_2_), 1673s (C=O), 1593w, 1458w, 1418w (C=C), 1333w ν_s_ (CNS), 1303vs, 1287s, 1235m ν_as_ (SO_2_), 1172vs, 1156vs ν_s_ (SO_2_), 1141m, 1124m, 1057w, 1020w, 970vs ν_as_ (CNS), 918w, 870w, 834w, 790m, 754s, 748s (γ CH), 717w, 677s γ (ring-Ph), 595vs, 564s, 539s, 521s, 469m, 417w. ^1^H-NMR (DMSOd_6_, 400 MHz): δ 7.92–7.73 (m, 4H, sac), 5.84 (sd, 2H, 2J(Pt−H) = 36 Hz, H_5,6cod_), 5.08 (sd, 2H, 2J(Pt−H) = 64 Hz, H_1,2cod_), 2.76–2.09 (m, 8H, H_3,4,7,8cod_). ^13^C NMR (DMSO-d_6_, 100 MHz): δ 159.1 (C_7_-sac), 141.7 (C_1_- sac), 137.2 (C_3_-sac), 135.5 (C_6_-sac), 134.4 (C_4_-sac), 124.4 (C_5_-sac), 121.0 (C_2_-sac), 106.2 (s, CH-COD) 101.8 (s, CH-COD), 30.7 (CH_2_-COD), 27.8 (CH_2_-COD). Molar conductivity, ΛM (MeOH, 298 K, 10^-3^ M) 6.1 S cm^2^mol^-1^ (nonelectrolyte). ESI–MS (m/z) : 696.5 (19%, calcd. 696.6) [Pt(sac)_2_(MeCN)_2_(MeOH) + Na]^+^, 598.0 (49%, calcd. 598.1) [Pt(sac)(COD)(MeOH)(MeCN)_2_]^+^, 522.2 (22%, calcd. 521.9) [M + 1]^+^, 408.4 (100%, calcd. 408.9) [Pt(sac)(MeOH)]^+^, 375.5 (32%, calcd. 376.0) [Pt(sac)]^+^, 333.9 (18%, calcd. 334.1) [Pt(COD)(MeOH) – H]^+^.

#### 6.2.2. Synthesis of [Pt(sac)_2_(COD)] (2)

The complex was prepared using a similar method explained for 1. The [PtCl_2_(COD)]/Na(sac)•2H_2_O ratio was 1/5 in this case. The complex was crystallized from a mixture of EtOH/H_2_O/DMSO after 4 weeks at rt.

[Pt(sac)_2_(COD)] (2). White powder. Yield: 159 mg (95%). Mp: 254–259 °C (decomp.). Anal. calcd. for C_22_H_20_N_2_O_6_PtS_2_: C, 39.58; H, 3.02; N, 4.20%. Found C, 39.42; H, 3.33; N, 4.48%. IR (ν /cm^-1^): 3077w, 3022w (C=CH), 2919w (CH_2_), 1691s (C=O), 1595w, 1459w, 1424w (C=C), 1331w ν_s_ (CNS), 1301vs, 1283s, 1233s ν_as_ (SO_2_), 1170vs, 1156vs ν_s_ (SO_2_), 1140s, 1121m, 1081w, 1058w, 1013w, 969vs ν_as_ (CNS), 870w, 834w, 789m, 747vs (γ CH), 716w, 675vs γ (ring-Ph), 594vs, 561s, 539s, 520vs, 463m, 416w. ^1^H-NMR (DMSO-d_6_, 400 MHz): δ 7.94–7.77 (m, 8H, sac), 5.52 (m, 4H, ^2^J(Pt−H) = 40 Hz, H_1,2,5,6cod_), 2.78–2.08 (m, 8H, H_3,4,7,8cod_). ^13^C NMR (DMSO-d_6_, 100 MHz): δ 158.9 (C_7_-sac), 141.7 (C_1_-sac), 137.2 (C_3_-sac), 135.7 (C_6_-sac), 134.4 (C_4_-sac), 125.1 (C_5_-sac), 121.4 (C_2_-sac), 106.1 (s, CH-COD), 30.4 (CH_2_-COD). Molar conductivity, ΛM (MeOH, 298 K, 10^-3^M) 7.6 S cm^2^mol^-1^ (nonelectrolyte). ESI–MS (m/z) : 691.4 (39%, calcd. 691.0) [M + Na]^+^, 664.1 (13%, calcd. 664.5) [Pt(sac)_2_(MeCN)_2_ + Na]^+^, 597.7 (38%, calcd. 598.1) [Pt(sac)(COD)(MeOH)(MeCN)_2_]^+^, 408.5 (63%, calcd. 408.9) [Pt(sac)(MeOH)]^+^, 375.5 (100%, calcd. 376.0) [Pt(sac)]sup>+, 333.7 (52%, calcd. 334.1) [Pt(COD)(MeOH) – H]^+^.

### 6.3. X-ray crystallography

The intensity data of the platinum(II) complexes were collected on a Rigaku Xcalibur X-ray diffractometer with EOS CCD detector using Mo-Kα radiation (0.71073 Å) with ω-scan mode. The structures were solved by direct methods using SHELXT [34], and refined by a full-matrix least-squares minimization with SHELXL [35], using Olex2 [36]. All nonhydrogen atoms were refined anisotropically, while all hydrogen atoms were located at calculated positions and refined by using riding model. Crystals of 2 displayed twinning and therefore, its structure was refined as a 2-component twin with a transformation matrix of [-1 0 0 / 0 1 0 / 0 0 -1]. Using an HKLF4 refinement in SHELXL, the fractional contribution of the major twin component was refined to 0.54 (4). Details of the data collection and structure refinement are given in Table 2.

**Table 2:** Crystallographic data and structure refinement for 1 and 2.

	**1**	**2**
Empirical formula	C_15_H_16_ClNO_3_PtS	C_22_H_20_N_2_O_6_PtS_2_
Formula weight	520.89	667.61
Crystal system	monoclinic	orthorhombic
Space group	P2_1_/c	Pna 2_1_
a, Å	8.1776(3)	19.9229(8)
b, Å	12.1960(5)	8.8086(3)
c, Å	16.1270(5)	12.5704(5)
α, deg	90	90
β, deg	95.445(3)	90
γ, deg	90	90
V, Å^3^	1601.15(10)	2206.02 (15)
T, K	295(2)	293(2)
Z	4	4
ρ_calc_(g cm^-3^)	2.161	2.010
μ (mm^-1^)	9.071	6.594
F(000)	992	1296
θ (°)	3.009--25.678	3.003-25.025
Collected refls	5261	7622
Data/parameters	3011/200	2911/337
Goodness-of-fit	1.012	1.035
R_1_ [I> 2σ]	0.0265	0.0636
wR_2_	0.0459	0.1747
CCDC number	1981760	1981761

## Supplementary data

Crystallographic data and refinement parameters of 1 and 2 have been deposited at the Cambridge Crystallographic Data Centre with CCDC numbers 1981760 and 1981761, respectively. These data can be obtained free of charge via www.ccdc.cam.ac.uk/data_request/cif, or by emailing data_request@ccdc.cam.ac.uk, or by contacting The Cambridge Crystallographic Data Centre.
